# New onset autoimmune disease following a SARS-CoV-2 infection: A systematic review protocol

**DOI:** 10.1371/journal.pone.0335766

**Published:** 2025-10-30

**Authors:** Alicia A. Grima, Linda T. Hiraki, Shelly Bolotin, Andrew D. Paterson, Jennifer D. Brooks

**Affiliations:** 1 Dalla Lana School of Public Health, University of Toronto, Toronto, Ontario, Canada; 2 Program in Genetics and Genome Biology, The Hospital for Sick Children, Toronto, Ontario, Canada; 3 Division of Rheumatology, The Hospital for Sick Children, Toronto, Ontario, Canada; 4 Public Health Ontario, Toronto, Ontario, Canada; 5 Department of Laboratory Medicine and Pathobiology, University of Toronto, Toronto, Ontario, Canada; University of Yaounde I Faculty of Medicine and Biomedical Sciences: Universite de Yaounde I Faculte de Medecine et des Sciences Biomedicales, CAMEROON

## Abstract

Severe acute respiratory syndrome coronavirus 2 (SARS-CoV-2) has infected millions of people worldwide. While most infected individuals who survive do so with no long-term consequences, approximately 10 to 70% develop long-term sequelae. Of particular concern has been the development of autoimmune diseases. Viral triggers for autoimmune disease have been thoroughly studied for previous viral infections and several recent studies have sought to investigate the link between SARS-CoV-2 and new onset autoimmune disease. Several reviews have also been conducted on the topic, however, many of these reviews are limited in focus, emphasizing biological mechanisms and case reports, as opposed to estimates of risk. Further, these reviews do not capture more recent cohort studies that have been published investigating the association between SARS-CoV-2 and new onset autoimmune disease. Therefore, there is a need for a more comprehensive and temporally updated systematically conducted review of the literature to address the question *What is the risk of incident (i.e., new onset) autoimmune disease following a SARS-CoV-2 infection among adults (≥18 years)?.* A systematic search of MEDLINE, EMBASE, CINAHL, and grey literature will be conducted, with results screened in duplicate in two stages: 1) Title and abstract screening and 2) Full text screening. A standardized data extraction sheet will be used on any studies passing through both stages of screening to extract details on publication, study population, exposure, and outcomes. Narrative and tabular synthesis of overall findings will be conducted, with diversity and heterogeneity of included studies discussed. If possible, a meta-analysis will also be conducted to combine findings of risk across the included studies. This protocol has been registered to PROSPERO (registration number: CRD42024594446).

## Introduction

Severe acute respiratory syndrome coronavirus 2 (SARS-CoV-2) has infected millions of people worldwide, causing over 770 million reported cases of coronavirus disease 2019 (COVID-19) and over 7 million reported deaths to date [1], although these numbers may represent a large underestimate of the true burden of the pandemic [2–4]. In Canada there have been over 4.7 million confirmed cases and over 50 thousand confirmed deaths to date [1]. While the majority of infected individuals who survive do so with no long-term consequences, approximately 10–70% develop long-term sequelae [5,6]. Of particular concern has been the development of autoimmune diseases following a SARS-CoV-2 infection.

In general, autoimmune diseases arise when the body does not recognize its own cells and tissues resulting in an immune response against itself [7]. This ultimately leads to damage in the targeted tissue. Autoimmune diseases are not specific to one area of the body, instead this self-directed immune response can occur in any number of organ systems, and as such, there are many different autoimmune disease types [7]. For example, rheumatoid arthritis is an inflammatory autoimmune disease which leads to destruction of the diarthrodial joints [8]. Type 1 diabetes however, results from a self-targeted immune response destroying pancreatic B-cells [8].

Studies have uncovered complex underlying causes of autoimmune diseases, including genetic predispositions (often a combination of several genes) and environmental factors (including chemical exposure, diet, and viral infections) [9,10]. Viral triggers for autoimmune disease, in particular, have been thoroughly studied with numerous examples documented [11–13] and three mechanisms for this phenomenon proposed [10,14].

Several recent studies have sought to investigate the link between SARS-CoV-2 and new onset autoimmune disease [15–18]. The authors of one such study were able to conduct a large retrospective cohort study utilizing global COVID-19 data [15]. They looked at new diagnoses of various autoimmune conditions among those with and without a history of COVID-19 infection. Associated risks of the majority of conditions they investigated were higher among those who had a history of a COVID-19 infection compared to those who did not, including the risks of rheumatoid arthritis, systemic lupus erythematosus, and psoriasis [15].

Several reviews have also been conducted on new onset autoimmune disease following SARS-CoV-2 infection [19–23]. Many of these reviews however are limited in focus, emphasizing biological mechanisms and case reports, as opposed to estimates of risk. For example, the review by Liu et al. is mainly mechanistic with some cases of autoimmune disease [19]. Alternatively, these reviews focus on specific autoimmune disease subtypes rather than autoimmune disease as a whole. For example, Gracia-Ramos et al. presented findings on new onset rheumatic autoimmune diseases [22] and Nabizadeh et al. on autoimmune encephalitis [20]. Further, these reviews do not capture more recent cohort studies that have been published investigating the association between SARS-CoV-2 and new onset autoimmune disease, such as those by Chang et al., Lim et al., Peng et al., Tesch et al., and Hileman et al. [15–18,24]. Therefore, there is a need for a more comprehensive and temporally updated systematically conducted review of the literature.

The objective of this systematic review is to answer the question: *What is the risk of incident (i.e., new onset) autoimmune disease following a SARS-CoV-2 infection among adults (≥18 years)?* Additionally, this review will evaluate the quality of this evidence.

## Methods

The Preferred Reporting Items for Systematic review and Meta-Analysis Protocols (PRISMA-P) 2015 checklist was used to guide the development of this protocol (Table in S1 Table).

### Eligibility criteria

As per Joanna Briggs Institute guidance for risk/etiology reviews, the **P**opulation, **E**xposure, and **O**utcome, or PEO, framework will be used as the basis of determining study inclusion criteria for this review [25,26].

### Population

The population for this review will be any individuals 18 years of age or older (i.e., adults). Individuals are not required to have a particular condition as the review will be focused on new onset autoimmune disease. Studies focusing solely on a pediatric population will be excluded from this review. The reasoning for this is that pediatrics are a distinct patient population, with autoimmune diseases being rarer in children [27]. The clinical course of an autoimmune disease may also differ between children and adults [28]. If a potential study for inclusion includes both children and adults, the study would only be included if it provides age stratified risk estimates.

### Exposure

The exposure for this review will be SARS-CoV-2 infection. Studies which do not have SARS-CoV-2 infection as the exposure will be excluded from this review. Any type of reported SARS-CoV-2 infection (i.e., self-report, polymerase chain reaction confirmed, positive antigen test, etc.) will be acceptable for the exposure. The reason for no test type restriction is that different testing methods were available at different points of the pandemic and availability of testing types also may have varied by place of residence (e.g., province/state, country, etc.). Thus, studies conducted in different regions or countries may have captured a positive test in a different way. This should allow our review to provide a more comprehensive overview of the literature, and test types will be noted in the narrative synthesis.

### Outcome

To be eligible for inclusion a study must report new onset autoimmune disease as its outcome. A list of autoimmune diseases was compiled from outcomes used in several recent cohort studies on the topic and reviewed by a rheumatologist (LTH) (Table in S2 Table). This list formed the basis of the search strategy, but any new onset autoimmune disease will be included in the review.

For a study to be relevant to this review and thus included, the outcome must occur following a SARS-CoV-2 infection. For example, if a study reported on autoimmune disease presentations of the infection itself, then the study would not be included in this review. If a different study reported on disease flare ups in rheumatoid arthritis patients following a SARS-CoV-2 infection, then this study would also not be included as it focuses on prevalent autoimmune disease. However, if a study reported on incident multiple sclerosis in individuals who have a history of SARS-CoV-2 infection then this study would be included. Both composite (i.e., overall autoimmune disease risk) and disease-specific outcomes are of interest for this review.

### Study timeline

Eligible studies will be restricted to publishing dates between December 2019 and present. This is to ensure capture of only studies related to the current COVID-19 pandemic and thus SARS-CoV-2 infections.

### Study design

The focus of this review is on autoimmune disease risk, as such case-reports, commentaries, and editorials will be excluded as these are not study designs which evaluate risk. Reviews will also be excluded but those relevant to the research question will be flagged for reference review.

### Language

No exclusions will be made on the basic of language. Non-English language studies will be translated to English and subsequently screened at the full text screening stage.

### Information sources

A literature search of MEDLINE, EMBASE, and CINAHL will be conducted. Grey Literature will also be searched using Canada’s Drug Agency Grey Matters resource [29]. Lastly, studies that were included in relevant reviews on the topic will also be screened to ensure no studies were missed by our search. As discussed above in *Eligibility Criteria*, the search will be restricted to December 2019 to present.

### Search strategy

The search included subject headings and keywords for SARS-CoV-2, post-COVID conditions and long COVID, and autoimmune diseases. Search terms for SARS-CoV-2 were based on search strings from Research Information Specialists at Canada’s Drug Agency [30], and search terms for long COVID were based on filters from the Geoffrey & Robyn Sperber Health Sciences Library, University of Alberta [31]. Search strategies for excluding animal studies were obtained from publicly available systematic review resources [32,33]. Lastly, search terms for autoimmune disease were based on a review by Coriddi et al. [34].

The full search strategies are available in the S3-S5 Files. There are many cases where antiquated, non-standard, exclusionary, and potentially offensive terms for the COVID-19 pandemic have been used in past and present literature [35]. In light of this, the authors have included such terms in the search strategies in order to conduct a sensitive, comprehensive search for relevant studies. The authors of this review recognize and acknowledge the inappropriate and harmful nature of these terms, and indicate that the full search strategies include them, so that the reader can determine how they would like to proceed [35].

### Study records

#### Data management.

Records identified through the literature search will be uploaded to Covidence, an online systematic review tool. Study records can be screened by multiple users within Covidence’s platform. Data extraction is also possible through Covidence. Screening pilots to ensure agreement between reviewers and data extraction will occur using Microsoft Excel.

#### Selection process.

All articles captured by the search will be screened in two stages: 1) Title and abstract screening and 2) Full text screening. Titles and abstracts will be screened by two independent reviewers, with any disagreements resolved through discussion or a third screener. Pilots in batches of 50 articles will be used to ensure agreement between reviewers reaches ≥75% prior to the start of formal screening. Articles that proceed to the full text screening stage will also be subject to screening by two independent reviewers and pilots will also be used to ensure reviewer agreement reaches ≥75%. Reasons for exclusion at the full text stage will be recorded for any excluded articles. A Preferred Reporting Items for Systematic reviews and Meta-Analyses (PRISMA) flow diagram will be created to summarize the study selection process [36]. Screening of articles is currently in progress and the below proposed analysis is anticipated to be completed by the end of 2025, with manuscript submission in the beginning of 2026.

#### Data collection process.

A standardized data extraction sheet will be developed and used for all articles passing through the second stage of article screening. Data extraction will either be conducted by two independent reviewers with discussion or a third reviewer to resolve conflicts, or by one reviewer followed by validation by a second independent reviewer. If more than one article presents the same study data, data from the most up to date article will be selected as the primary record and missing data supplemented from the other study record. Outcome measures will be extracted as presented in the source text (i.e., risk ratio, hazard ratio, rate, etc.).

### Data items

The data to be extracted from included studies will include:

- Publication details (e.g., publication year, etc.)
- Study details (e.g., study design, sample size, funding, etc.)
- Study population (e.g., age, race and ethnicity, etc.)
- Exposure details (e.g., type of test, infection severity, etc.)
- Outcomes (e.g., autoimmune disease types and counts, risk/measures of association, etc.) [37,38]

### Risk of bias in individual studies

All included articles will undergo critical appraisal at the study level by two independent reviewers, with any disagreements resolved through discussion or a third reviewer [37]. This critical appraisal will include an assessment of internal validity and risk of bias. Joanna Briggs Institute critical appraisal checklists appropriate to study design will be utilized to ensure the same standards are applied to each article [37]. Data synthesis will include a summary of the quality of evidence for new onset autoimmune diseases post SARS-CoV-2 infection.

### Data synthesis

Narrative and tabular synthesis of the data extracted from included studies will be conducted for this systematic review [26,37]. These will include synthesis of study characteristics, quality, and overall findings of the included studies. The clinical diversity (i.e., participants, outcomes, etc.) and methodological diversity (i.e., study design, outcome measurement, risk of bias, etc.) will be discussed [39]. Statistical heterogeneity of included studies will also be assessed using the Cochrane *Q* statistic (P < 0.10) and the *I*^*2*^ statistic with standard thresholds [38,39].

If possible (sufficient number of studies, reported measures of association, acceptable heterogeneity), a meta-analysis will be conducted to combine findings across the included studies [26,37]. A meta-analysis would be conducted for composite risk, disease-specific risk, or both.

Studies will be restricted to only those with test negative controls for a sensitivity analysis. A second sensitivity analysis will exclude all studies utilizing only rapid antigen tests from the analysis.

### Meta-bias(es)

A funnel plot will be produced, and asymmetry evaluated in order to investigate the potential for small-study effects [40].

### Confidence in cumulative evidence

In addition to evaluating the risk of bias for individual study outcomes (as described in *Risk of bias in individual studies*), the overall quality of the evidence will be assessed using the Grading of Recommendations Assessment, Development and Evaluation framework [41].

## Discussion

If any amendments to this protocol are required, changes will be noted on PROSPERO (registration number: CRD42024594446).

## Supporting information

S1 Table PRISMA-P (Preferred Reporting Items for Systematic review and Meta-Analysis Protocols) 2015 checklist: recommended items to address in a systematic review protocol*. (DOCX)

S2 Table Autoimmune disease outcomes and their respective International Classification of Diseases (ICD) −10 code(s). (DOCX)

S3 File Search strategy for MEDLINE. (DOCX)

S4 File Search strategy for EMBASE. (DOCX)

S5 File Search strategy for CINAHL. (DOCX)

## References

[1] World Health Organization. WHO Coronavirus (COVID-19) Dashboard. [cited 2025]. https://covid19.who.int/

[2] Lau H, Khosrawipour T, Kocbach P, Ichii H, Bania J, Khosrawipour V. Evaluating the massive underreporting and undertesting of COVID-19 cases in multiple global epicenters. Pulmonology. 2021;27(2):110–5. doi: 10.1016/j.pulmoe.2020.05.015 32540223 PMC7275155

[3] COVID-19 epidemiology update: Current situation. Government of Canada. 2024.

[4] Mathieu E, Ritchie H, Rodés-Guirao L, Appel C, Giattino C, Hasell J, et al. Coronavirus Pandemic (COVID-19) OurWorldInData.org. 2020. https://ourworldindata.org/coronavirus

[5] Greenhalgh T, Knight M, A’Court C, Buxton M, Husain L. Management of post-acute covid-19 in primary care. BMJ. 2020;370:m3026. doi: 10.1136/bmj.m3026 32784198

[6] Davis HE, McCorkell L, Vogel JM, Topol EJ. Long COVID: major findings, mechanisms and recommendations. Nat Rev Microbiol. 2023;21(3):133–46. doi: 10.1038/s41579-022-00846-2 36639608 PMC9839201

[7] Ngo ST, Steyn FJ, McCombe PA. Gender differences in autoimmune disease. Front Neuroendocrinol. 2014;35(3):347–69. doi: 10.1016/j.yfrne.2014.04.004 24793874

[8] Amador-Patarroyo MJ, Rodriguez-Rodriguez A, Montoya-Ortiz G. How does age at onset influence the outcome of autoimmune diseases?. Autoimmune Dis. 2012;2012:251730. doi: 10.1155/2012/251730 22195277 PMC3238350

[9] Makin S. Cracking the genetic code of autoimmune disease. Nature. 2021;595(7867):S57–9. doi: 10.1038/d41586-021-01839-6

[10] Sundaresan B, Shirafkan F, Ripperger K, Rattay K. The Role of Viral Infections in the Onset of Autoimmune Diseases. Viruses. 2023;15(3):782. doi: 10.3390/v15030782 36992490 PMC10051805

[11] Darmarajan T, Paudel KR, Candasamy M, Chellian J, Madheswaran T, Sakthivel LP, et al. Autoantibodies and autoimmune disorders in SARS-CoV-2 infection: pathogenicity and immune regulation. Environ Sci Pollut Res Int. 2022;29(36):54072–87. doi: 10.1007/s11356-022-20984-7 35657545 PMC9163295

[12] Finsterer J, Scorza FA. Guillain-Barre syndrome in 220 patients with COVID-19. Egypt J Neurol Psychiatr Neurosurg. 2021;57(1):55. doi: 10.1186/s41983-021-00310-7 33967575 PMC8094972

[13] Zhou S-Y, Zhang C, Shu W-J, Chong L-Y, He J, Xu Z, et al. Emerging Roles of Coronavirus in Autoimmune Diseases. Arch Med Res. 2021;52(7):665–72. doi: 10.1016/j.arcmed.2021.03.012 33875273 PMC8031002

[14] Smatti MK, Cyprian FS, Nasrallah GK, Al Thani AA, Almishal RO, Yassine HM. Viruses and Autoimmunity: A Review on the Potential Interaction and Molecular Mechanisms. Viruses. 2019;11(8):762. doi: 10.3390/v11080762 31430946 PMC6723519

[15] Chang R, Chen TY-T, Wang S-I, Hung Y-M, Chen H-Y, Wei C-CJ. Risk of autoimmune diseases in patients with COVID-19: A retrospective cohort study. EClinicalMedicine. 2023;56:101783. 36643619 10.1016/j.eclinm.2022.101783 PMC9830133

[16] Lim SH, Ju HJ, Han JH, Lee JH, Lee W-S, Bae JM, et al. Autoimmune and Autoinflammatory Connective Tissue Disorders Following COVID-19. JAMA Netw Open. 2023;6(10):e2336120. doi: 10.1001/jamanetworkopen.2023.36120 37801317 PMC10559181

[17] Peng K, Li X, Yang D, Chan SCW, Zhou J, Wan EYF, et al. Risk of autoimmune diseases following COVID-19 and the potential protective effect from vaccination: a population-based cohort study. EClinicalMedicine. 2023;63:102154. doi: 10.1016/j.eclinm.2023.102154 37637754 PMC10458663

[18] Tesch F, Ehm F, Vivirito A, Wende D, Batram M, Loser F, et al. Incident autoimmune diseases in association with SARS-CoV-2 infection: a matched cohort study. Clin Rheumatol. 2023;42(10):2905–14. doi: 10.1007/s10067-023-06670-0 37335408 PMC10497688

[19] Liu Y, Sawalha AH, Lu Q. COVID-19 and autoimmune diseases. Curr Opin Rheumatol. 2021;33(2):155–62. doi: 10.1097/BOR.0000000000000776 33332890 PMC7880581

[20] Nabizadeh F, Balabandian M, Sodeifian F, Rezaei N, Rostami MR, Naser Moghadasi A. Autoimmune encephalitis associated with COVID-19: A systematic review. Mult Scler Relat Disord. 2022;62:103795. doi: 10.1016/j.msard.2022.103795 35472834 PMC8983076

[21] Sgamato C, Rocco A, Compare D, Minieri S, Marchitto SA, Maurea S, et al. Autoimmune liver diseases and SARS-CoV-2. World J Gastroenterol. 2023;29(12):1838–51. doi: 10.3748/wjg.v29.i12.1838 37032727 PMC10080695

[22] Gracia-Ramos AE, Martin-Nares E, Hernández-Molina G. New Onset of Autoimmune Diseases Following COVID-19 Diagnosis. Cells. 2021;10(12):3592. doi: 10.3390/cells10123592 34944099 PMC8700122

[23] Dotan A, Muller S, Kanduc D, David P, Halpert G, Shoenfeld Y. The SARS-CoV-2 as an instrumental trigger of autoimmunity. Autoimmun Rev. 2021;20(4):102792. doi: 10.1016/j.autrev.2021.102792 33610751 PMC7892316

[24] Hileman CO, Malakooti SK, Patil N, Singer NG, McComsey GA. New-onset autoimmune disease after COVID-19. Front Immunol. 2024;15:1337406. doi: 10.3389/fimmu.2024.1337406 38390319 PMC10883027

[25] Munn Z, Stern C, Aromataris E, Lockwood C, Jordan Z. What kind of systematic review should I conduct? A proposed typology and guidance for systematic reviewers in the medical and health sciences. BMC Med Res Methodol. 2018;18(1):5. doi: 10.1186/s12874-017-0468-4 29316881 PMC5761190

[26] Moola S, Munn Z, Sears K, Sfetcu R, Currie M, Lisy K, et al. Conducting systematic reviews of association (etiology): The Joanna Briggs Institute’s approach. Int J Evid Based Healthc. 2015;13(3):163–9. doi: 10.1097/XEB.0000000000000064 26262566

[27] Hospital BC. Autoimmune Diseases. https://www.childrenshospital.org/conditions/autoimmune-diseases

[28] Tarr T, Dérfalvi B, Győri N, Szántó A, Siminszky Z, Malik A, et al. Similarities and differences between pediatric and adult patients with systemic lupus erythematosus. Lupus. 2015;24(8):796–803. doi: 10.1177/0961203314563817 25516474

[29] Canada’s Drug Agency. Grey Matters: A Tool for Searching Health-related Grey Literature Ottawa. 2024. https://greymatters.cda-amc.ca

[30] COVID-19 Search Strings: Canada’s Drug Agency. [Cited September 25, 2024]. https://www.cda-amc.ca/literature-searching-tools/covid-19-search-strings

[31] Campbell SM. Filter to Retrieve Studies Related to Long COVID in the OVID Medline Database: Geoffrey & Robyn Sperber Health Sciences Library, University of Alberta. [Cited January 10, 2023]. https://docs.google.com/document/d/1RrEgiFH1M1k_V-S-xBbXrMZrXwaAJ7RO0Baa-U_9dL0/edit#heading=h.v97m5qxqnbli

[32] Search Filters/Hedges: American University of Beirut: University Libraries Library Guides. [Cited July 23, 2024]. https://aub.edu.lb.libguides.com/c.php?g=329862&p=3023731

[33] McGill University Health Centre Libraries. Excluding Animal Studies 2019. https://www.bibliothequescusm.ca/Documents/TIPS_Excluding_Animals_FINAL_EN-20190724.pdf

[34] Coriddi M, Burke EA, Myers P, Soudant C, McCarthy CM. Autoimmune Disease and Breast Implants: Systematic Review of Outcomes. Ann Plast Surg. 2023;90(4):385–8. doi: 10.1097/SAP.0000000000002930 34117137 PMC8660949

[35] Townsend W, Anderson P, Capellari E, Haines K, Hansen S, James L. Addressing antiquated, non-standard, exclusionary, and potentially offensive terms in evidence syntheses and systematic searches. University of Michigan Library; 2022.

[36] Page MJ, McKenzie JE, Bossuyt PM, Boutron I, Hoffmann TC, Mulrow CD, et al. The PRISMA 2020 statement: an updated guideline for reporting systematic reviews. BMJ. 2021;372:n71. doi: 10.1136/bmj.n71 33782057 PMC8005924

[37] Aromataris E, Munn Z. JBI manual for evidence synthesis. Aromataris E, Munn Z, editors. JBI; 2020.

[38] Hamel C, Stevens A, Singh K, Ansari MT, Myers E, Ziegler P, et al. Do sugar-sweetened beverages cause adverse health outcomes in adults? A systematic review protocol. Syst Rev. 2014;3:108. doi: 10.1186/2046-4053-3-108 25248499 PMC4178316

[39] Higgins JPT, Thomas J, Chandler J, Cumpston M, Li T, Page MJ, et al. Analysing data and undertaking meta-analyses. Cochrane Handbook for Systematic Reviews of Interventions. Cochrane; 2023.

[40] Higgins JPT, Thomas J, Chandler J, Cumpston M, Li T, Page M. Assessing risk of bias due to missing evidence in a meta-analysis. Cochrane Handbook for Systematic Reviews of Interventions. Cochrane; 2024. p. 40.

[41] Schünemann HJ, Higgins JPT, Vist GE, Glasziou P, Akl EA, Skoetz N. Completing ‘Summary of findings’ tables and grading the certainty of the evidence. In: Higgins JPT, Thomas J, Chandler J, Cumpston M, Li T, Page MJ, editors. Cochrane Handbook for Systematic Reviews of Interventions. Cochrane; 2024.

