# Supplementary material for: New onset autoimmune disease following a SARS-CoV-2 infection: A systematic review protocol
Source: PLoS One. 2025 Oct 30;20(10):e0335766. doi: 10.1371/journal.pone.0335766 (PMC12574822; doi:10.1371/journal.pone.0335766)
Supplement: S2 Table — (DOCX) [file pone.0335766.s002.docx]

**S2 Table*.* Autoimmune disease outcomes and their respective International Classification of Diseases (ICD) -10 code(s)**

| **Autoimmune Disease** | **ICD-10 Code(s)** | **Reference** |
| --- | --- | --- |
| Autoimmune hemolytic anemia | D59.1 | Tesch et al., 2023^18^ |
| Idiopathic thrombocytopenic purpura | D69.3 | Tesch et al., 2023^18^ |
| Cryoglobulinemia | D89.1 | Tesch et al., 2023^18^ |
| Autoimmune thyroid disease   - Grave’s disease - Hashimoto’s thyroiditis | E05, E06.2, E06.3 | Tesch et al., 2023^18^ |
| Type 1 diabetes mellitus | E10 | Chang et al., 2023; Tesch et al., 2023^15,18^ |
| Addison’s disease (or adrenal insufficiency) | E27.1 | Tesch et al., 2023^18^ |
| Multiple sclerosis | G35, G36, G37 | Tesch et al., 2023^18^ |
| Guillain-Barré-syndrome | G61.0 | Tesch et al., 2023^18^ |
| Myasthenia gravis | G70.0 | Tesch et al., 2023^18^ |
| Inflammatory bowel disease   - Crohn’s disease (or Morbus Crohn) - Ulcerative colitis | K50-52 | Chang et al., 2023^15^; Tesch et al., 2023^18^; Lim et al., 2023^16^ |
| Biliary cholangitis | K74.3 | Tesch et al., 2023^18^ |
| Autoimmune hepatitis | K75.4 | Tesch et al., 2023^18^ |
| Celiac disease | K90.0 | Chang et al., 2023; Tesch et al., 2023^15,18^ |
| Pemphigus vulgaris | L10.0, L10.2, L10.2, L10.3, L10.4 | Tesch et al., 2023^18^ |
| Bullous pemphigoid | L12 | Tesch et al., 2023^18^ |
| Dermatitis herpetiformis Duhring | L13.0 | Tesch et al., 2023^18^ |
| Psoriasis | L40 | Chang et al., 2023; Tesch et al., 2023; Lim et al., 2023^15,16,18^ |
| Alopecia   - Alopecia areata - Alopecia totalis | L63 | Tesch et al., 2023; Lim et al., 2023^16,18^ |
| Vitiligo | L80 | Tesch et al., 2023; Lim et al., 2023^16,18^ |
| Cutaneous lupus erythematosus | L93 | Tesch et al., 2023^18^ |
| Rheumatoid arthritis | M05, M06, M08, M12.3 | Chang et al., 2023; Tesch et al., 2023^15,18^ |
| Adult-onset Still disease | M06.1 | Lim et al., 2023^16^ |
| Vasculitis   - Polyarteritis nodosa - Anti-neutrophil cytoplasmic antibody (ANCA)-associated vasculitis (AAV) - Goodpasture syndrome - Takayasu arteritis - Arteritis temporalis (or giant cell arteritis or temporal arteritis or cranial arteritis) | M20-31 or L95 | Chang et al., 2023^15^; Tesch et al., 2023^18^; Lim et al., 2023^16^ |
| Systemic lupus erythematosus | M32 | Chang et al., 2023; Tesch et al., 2023; Lim et al., 2023^15,16,18^ |
| Dermatopolymyositis   - Dermatomyositis - Polymyositis | M33 | Chang et al., 2023^15^; Tesch et al., 2023; Lim et al., 2023^16,18^ |
| Systemic sclerosis (or systemic scleroderma) | M34 | Tesch et al., 2023^18^; Chang et al., 2023^15^; Lim et al., 2023^16^ |
| Sjögren’s syndrome | M35.0 | Chang et al., 2023; Tesch et al., 2023; Lim et al., 2023^15,16,18^ |
| Mixed connective tissue disease | M35.1 | Chang et al., 2023^15^ |
| Polymyalgia rheumatica | M35.3 | Chang et al., 2023; Tesch et al., 2023^15,18^ |
| Ankylosing spondylitis | M45 | Chang et al., 2023; Tesch et al., 2023; Lim et al., 2023^15,16,18^ |
