# Supplementary material for: New onset autoimmune disease following a SARS-CoV-2 infection: A systematic review protocol
Source: PLoS One. 2025 Oct 30;20(10):e0335766. doi: 10.1371/journal.pone.0335766 (PMC12574822; doi:10.1371/journal.pone.0335766)
Supplement: S3 File — (DOCX) [file pone.0335766.s003.docx]

**S3 File. Search strategy for MEDLINE**

1. COVID-19/ or SARS-CoV-2/ or Severe Acute Respiratory Syndrome/ or Severe acute respiratory syndrome-related coronavirus/
2. (coronavirus/ or betacoronavirus/ or coronavirus infections/) and (disease outbreaks/ or epidemics/ or pandemics/)
3. (COVID-19 or SARS-CoV-2 or Severe Acute Respiratory Syndrome or coronavirus or nCoV* or 2019nCoV or 19nCoV or COVID19* or COVID or SARS-COV-2 or SARSCOV-2 or SARS-COV2 or SARSCOV2 or SARS coronavirus 2).tw,kf.
4. (nCoV* or 2019nCoV or 19nCoV or COVID19* or COVID or SARS-COV-2 or SARSCOV-2 or SARS-COV2 or SARSCOV2 or SARS coronavirus 2 or Severe Acute Respiratory Syndrome Coronavirus 2 or Severe Acute Respiratory Syndrome Corona Virus 2).tw,kf.
5. ((new or novel or "19" or "2019" or Wuhan or Hubei or China or Chinese) adj3 (coronavirus* or corona virus* or betacoronavirus* or CoV or HCoV)).tw,kf.
6. ((coronavirus* or corona virus* or betacoronavirus*) adj3 (pandemic* or epidemic* or outbreak* or crisis)).tw,kf.
7. ((Wuhan or Hubei) adj5 pneumonia).tw,kf.
8. 1 or 2 or 3 or 4 or 5 or 6 or 7
9. Post-Acute COVID-19 Syndrome/ or Post-Infectious Disorders/
10. ((post-acute adj2 COVID-19 adj2 syndrome) or (post-infectious adj2 disorder) or (long adj5 COVID) or PASC or Post-Acute Sequalae of COVID-19 or (post-COVID adj5 syndrome) or (Post-COVID adj6 Condition$)).tw,kf.
11. ((Covid or Covid19 or "corona virus 2019" or "coronavirus 2019" or SARS-CoV-2 or "B.1.1.7" or "B.1.351" or "B.1.1.28" or "B.1.617" or "BA.1" or "BA.2" or "BA.3" or "BA.4" or "BA.5" or omicron or deltacron or "delta variant" or "delta subvariant" or "XBB.1.3") adj3 (prolonged or "long haul*" or chronic or lingering or ongoing or persistent or "long term" or "more than 12 weeks" or "more than 24 weeks")).tw,kf.
12. 9 or 10 or 11
13. 8 or 12
14. Autoimmune Diseases/ or ((autoimmun* or auto immun*) and (disease* or disorder*)).tw,kf.
15. Anemia, Hemolytic, Autoimmune/ or Autoimmune hemolytic an?emia*.tw,kf.
16. Purpura, Thrombocytopenic, Idiopathic/ or Idiopathic thrombocytopenic purpura*.tw,kf.
17. Cryoglobulinemia/ or Cryoglobulinemia*.tw,kf.
18. Thyroiditis, Autoimmune/ or Graves Disease/ or Hashimoto Disease/ or (Autoimmune Thyroiditi* or Autoimmune thyroid disease* or Grave$ disease* or Hashimoto$ Disease* or Hashimoto$ thyroiditi*).tw,kf.
19. Diabetes Mellitus, Type 1/ or (Type 1 adj4 diabet*).tw,kf.
20. Addison Disease/ or Adrenal Insufficiency/ or (Addison$ disease* or Adrenal insufficienc*).tw,kf.
21. Multiple Sclerosis/ or Multiple scleros*.tw,kf.
22. Guillain-Barre Syndrome/ or Guillain-Barre-Syndrome*.tw,kf.
23. Myasthenia Gravis/ or Myasthenia Gravis.tw,kf.
24. Inflammatory Bowel Diseases/ or Colitis, Ulcerative/ or Crohn Disease/ or (Inflammatory Bowel Disease* or Ulcerative coliti* or Crohn$ Disease* or Morbus Crohn*).tw,kf.
25. Liver Cirrhosis, Biliary/ or Biliary cholangiti*.tw,kf.
26. Hepatitis, Autoimmune/ or Autoimmune hepatiti*.tw,kf.
27. Celiac Disease/ or C?eliac disease*.tw,kf.
28. Pemphigus/ or Pemphigus vulgaris.tw,kf.
29. Pemphigoid, Bullous/ or Bullous pemphigoid*.tw,kf.
30. Dermatitis Herpetiformis/ or (Dermatitis herpetiform* or Duhring$ disease*).tw,kf.
31. Psoriasis/ or Psorias*.tw,kf.
32. Alopecia/ or Alopecia Areata/ or Alopecia*.tw,kf.
33. Vitiligo/ or Vitiligo*.tw,kf.
34. Lupus Erythematosus, Cutaneous/ or Cutaneous lupus erythemato*.tw,kf.
35. Arthritis, Rheumatoid/ or Rheumatoid arthriti*.tw,kf.
36. Still's Disease, Adult-Onset/ or Adult-onset Still$ disease*.tw,kf.
37. Vasculitis/ or Anti-Neutrophil Cytoplasmic Antibody-Associated Vasculitis/ or Polyarteritis Nodosa/ or Anti-Glomerular Basement Membrane Disease/ or Takayasu Arteritis/ or Giant Cell Arteritis/ or (Vasculiti* or Anti-Neutrophil Cytoplasmic Antibody-Associated Vasculiti* or ANCA-Associated Vasculiti* or Polyarteritis Nodos* or Anti-Glomerular Basement Membrane Disease* or Goodpasture$ syndrome* or Takayasu arteriti* or Arteritis temporalis or Giant cell arteriti* or Temporal arteriti* or Cranial arteriti*).tw,kf.
38. Lupus Erythematosus, Systemic/ or Systemic lupus erythemato*.tw,kf.
39. Dermatomyositis/ or Polymyositis/ or (Dermatopolymyositi* or Dermatomyositi* or Polymyositi*).tw,kf.
40. Scleroderma, Systemic/ or Systemic sclero*.tw,kf.
41. Sjogren's Syndrome/ or Sjogren$ syndrome*.tw,kf.
42. Mixed Connective Tissue Disease/ or Mixed connective tissue disease*.tw,kf.
43. Polymyalgia Rheumatica/ or Polymyalgia rheumatica.tw,kf.
44. Spondylitis, Ankylosing/ or Ankylosing spondylit*.tw,kf.
45. 14 or 15 or 16 or 17 or 18 or 19 or 20 or 21 or 22 or 23 or 24 or 25 or 26 or 27 or 28 or 29 or 30 or 31 or 32 or 33 or 34 or 35 or 36 or 37 or 38 or 39 or 40 or 41 or 42 or 43 or 44
46. 13 and 45
47. limit 46 to yr="2019 -Current"
48. 47 not (exp animals/ not humans.sh.)
