# Supplementary material for: New onset autoimmune disease following a SARS-CoV-2 infection: A systematic review protocol
Source: PLoS One. 2025 Oct 30;20(10):e0335766. doi: 10.1371/journal.pone.0335766 (PMC12574822; doi:10.1371/journal.pone.0335766)
Supplement: S4 File — (DOCX) [file pone.0335766.s004.docx]

**S4 File. Search strategy for EMBASE**

1. severe acute respiratory syndrome coronavirus 2/ or sars-related coronavirus/ or coronavirus disease 2019/ or coronavirus infection/ or severe acute respiratory syndrome/ or severe acute respiratory syndrome coronavirus 2/
2. (coronavirinae/ or betacoronavirus/ or coronavirus infection/) and (epidemic/ or pandemic/)
3. (COVID-19 or SARS-CoV-2 or Severe Acute Respiratory Syndrome or coronavirus or nCoV* or 2019nCoV or 19nCoV or COVID19* or COVID or SARS-COV-2 or SARSCOV-2 or SARS-COV2 or SARSCOV2 or SARS coronavirus 2).tw,kw.
4. (nCoV* or 2019nCoV or 19nCoV or COVID19* or COVID or SARS-COV-2 or SARSCOV-2 or SARS-COV2 or SARSCOV2 or SARS coronavirus 2 or Severe Acute Respiratory Syndrome Coronavirus 2 or Severe Acute Respiratory Syndrome Corona Virus 2).tw,kw.
5. ((new or novel or "19" or "2019" or Wuhan or Hubei or China or Chinese) adj3 (coronavirus* or corona virus* or betacoronavirus* or CoV or HCoV)).tw,kw.
6. ((coronavirus* or corona virus* or betacoronavirus*) adj3 (pandemic* or epidemic* or outbreak* or crisis)).tw,kw.
7. ((Wuhan or Hubei) adj5 pneumonia).tw,kw.
8. 1 or 2 or 3 or 4 or 5 or 6 or 7
9. long covid/ or post-infectious syndrome/ or post-infectious syndrome/ or post infection complication/
10. ((post-acute adj2 COVID-19 adj2 syndrome) or (post-infectious adj2 disorder) or (long adj5 COVID) or PASC or Post-Acute Sequalae of COVID-19 or (post-COVID adj5 syndrome) or (Post-COVID adj6 Condition$)).tw,kw.
11. ((Covid or Covid19 or "corona virus 2019" or "coronavirus 2019" or SARS-CoV-2 or "B.1.1.7" or "B.1.351" or "B.1.1.28" or "B.1.617" or "BA.1" or "BA.2" or "BA.3" or "BA.4" or "BA.5" or omicron or deltacron or "delta variant" or "delta subvariant" or "XBB.1.3") adj3 (prolonged or "long haul*" or chronic or lingering or ongoing or persistent or "long term" or "more than 12 weeks" or "more than 24 weeks")).tw,kw.
12. 9 or 10 or 11
13. 8 or 12
14. autoimmune disease/ or ((autoimmun* or auto immun*) and (disease* or disorder*)).tw,kw.
15. autoimmune hemolytic anemia/ or Autoimmune hemolytic an?emia*.tw,kw.
16. idiopathic thrombocytopenic purpura/ or Idiopathic thrombocytopenic purpura*.tw,kw.
17. cryoglobulinemia/ or Cryoglobulinemia*.tw,kw.
18. autoimmune thyroiditis/ or graves disease/ or autoimmune hyperthyroidism/ or Hashimoto disease/ or (Autoimmune Thyroiditi* or Autoimmune thyroid disease* or Grave$ disease* Hashimoto$ Disease* or Hashimoto$ thyroiditi*).tw,kw.
19. insulin dependent diabetes mellitus/ or (Type 1 adj4 diabet*).tw,kw.
20. Addison disease/ or adrenal insufficiency/ or (Addison$ disease* or Adrenal insufficienc*).tw,kw.
21. multiple sclerosis/ or Multiple scleros*.tw,kw.
22. Guillain Barre syndrome/ or Guillain-Barre-Syndrome*.tw,kw.
23. myasthenia gravis/ or Myasthenia Gravis.tw,kw.
24. inflammatory bowel disease/ or Crohn disease/ or ulcerative colitis/ or (Inflammatory Bowel Disease* or Ulcerative coliti* or Crohn$ Disease* or Morbus Crohn*).tw,kw.
25. primary biliary cirrhosis/ or Biliary cholangiti*.tw,kw.
26. autoimmune hepatitis/ or Autoimmune hepatiti*.tw,kw.
27. celiac disease/ or C?eliac disease*.tw,kw.
28. pemphigus vulgaris/ or Pemphigus vulgaris.tw,kw.
29. bullous pemphigoid/ or Bullous pemphigoid*.tw,kw.
30. dermatitis herpetiformis/ or (Dermatitis herpetiform* or Duhring$ disease*).tw,kw.
31. psoriasis/ or Psorias*.tw,kw.
32. alopecia areata/ or alopecia/ or Alopecia*.tw,kw.
33. vitiligo/ or Vitiligo*.tw,kw.
34. skin lupus erythematosus/ or Cutaneous lupus erythemato*.tw,kw.
35. rheumatoid arthritis/ or Rheumatoid arthriti*.tw,kw.
36. adult onset Still disease/ or Adult-onset Still$ disease*.tw,kw.
37. vasculitis/ or ANCA associated vasculitis/ or polyarteritis nodosa/ or Goodpasture syndrome/ or aortic arch syndrome/ or giant cell arteritis/ or temporal arteritis/ or (Vasculiti* or Anti-Neutrophil Cytoplasmic Antibody-Associated Vasculiti* or ANCA-Associated Vasculiti* or Polyarteritis Nodos* or Anti-Glomerular Basement Membrane Disease* or Goodpasture$ syndrome* or Takayasu arteriti* or Arteritis temporalis or Giant cell arteriti* or Temporal arteriti* or Cranial arteriti*).tw,kw.
38. systemic lupus erythematosus/ or lupus erythematosus/ or Systemic lupus erythemato*.tw,kw.
39. dermatomyositis/ or polymyositis/ or (Dermatopolymyositi* or Dermatomyositi* or Polymyositi*).tw,kw.
40. systemic sclerosis/ or Systemic sclero*.tw,kw.
41. Sjoegren syndrome/ or Sjogren$ syndrome*.tw,kw.
42. mixed connective tissue disease/ or Mixed connective tissue disease*.tw,kw.
43. rheumatic polymyalgia/ or Polymyalgia rheumatica.tw,kw.
44. ankylosing spondylitis/ or Ankylosing spondylit*.tw,kw.
45. 14 or 15 or 16 or 17 or 18 or 19 or 20 or 21 or 22 or 23 or 24 or 25 or 26 or 27 or 28 or 29 or 30 or 31 or 32 or 33 or 34 or 35 or 36 or 37 or 38 or 39 or 40 or 41 or 42 or 43 or 44
46. 13 and 45
47. limit 46 to yr="2019 -Current"
48. 47 not ((exp animal/ or nonhuman/) not exp human/)
