# Supplementary material for: New onset autoimmune disease following a SARS-CoV-2 infection: A systematic review protocol
Source: PLoS One. 2025 Oct 30;20(10):e0335766. doi: 10.1371/journal.pone.0335766 (PMC12574822; doi:10.1371/journal.pone.0335766)
Supplement: S5 File — (DOCX) [file pone.0335766.s005.docx]

**S5 File. Search strategy for CINAHL**

1. (MH "COVID-19") OR (MH "COVID-19 Pandemic") OR (MH "SARS-CoV-2") OR (MH "Severe Acute Respiratory Syndrome") OR (MH "SARS Virus")
2. (MH "Coronavirus") OR (MH "Coronavirus Infections")
3. (MH "Disease Outbreaks")
4. S2 AND S3
5. TI (COVID-19 or SARS-CoV-2 or Severe Acute Respiratory Syndrome or coronavirus or nCoV* or 2019nCoV or 19nCoV or COVID19* or COVID or SARS-COV-2 or SARSCOV-2 or SARS-COV2 or SARSCOV2 or SARS coronavirus 2) OR AB (COVID-19 or SARS-CoV-2 or Severe Acute Respiratory Syndrome or coronavirus or nCoV* or 2019nCoV or 19nCoV or COVID19* or COVID or SARS-COV-2 or SARSCOV-2 or SARS-COV2 or SARSCOV2 or SARS coronavirus 2)
6. TI ( nCoV* or 2019nCoV or 19nCoV or COVID19* or COVID or SARS-COV-2 or SARSCOV-2 or SARS-COV2 or SARSCOV2 or SARS coronavirus 2 or Severe Acute Respiratory Syndrome Coronavirus 2 or Severe Acute Respiratory Syndrome Corona Virus 2 ) OR AB ( nCoV* or 2019nCoV or 19nCoV or COVID19* or COVID or SARS-COV-2 or SARSCOV-2 or SARS-COV2 or SARSCOV2 or SARS coronavirus 2 or Severe Acute Respiratory Syndrome Coronavirus 2 or Severe Acute Respiratory Syndrome Corona Virus 2 )
7. TI ( (new or novel or "19" or "2019" or Wuhan or Hubei or China or Chinese) N2 (coronavirus* or corona virus* or betacoronavirus* or CoV or HCoV) ) OR AB ( (new or novel or "19" or "2019" or Wuhan or Hubei or China or Chinese) N2 (coronavirus* or corona virus* or betacoronavirus* or CoV or HCoV) )
8. TI ( (coronavirus* or corona virus* or betacoronavirus*) N2 (pandemic* or epidemic* or outbreak* or crisis) ) OR AB ( (coronavirus* or corona virus* or betacoronavirus*) N2 (pandemic* or epidemic* or outbreak* or crisis) )
9. TI ( (Wuhan or Hubei) N4 pneumonia ) OR AB ( (Wuhan or Hubei) N4 pneumonia )
10. S1 OR S4 OR S5 OR S6 OR S7 OR S8 OR S9
11. (MH "Post-Acute COVID-19 Syndrome") OR (MH "Post-Infectious Disorders")
12. TI ( (post-acute N1 COVID-19 N1 syndrome) or (post-infectious N1 disorder) or (long N4 COVID) or PASC or Post-Acute Sequalae of COVID-19 or (post-COVID N4 syndrome) or (Post-COVID N5 Condition*) ) OR AB ( (post-acute N1 COVID-19 N1 syndrome) or (post-infectious N1 disorder) or (long N4 COVID) or PASC or Post-Acute Sequalae of COVID-19 or (post-COVID N4 syndrome) or (Post-COVID N5 Condition*) )
13. TI ( (Covid or Covid19 or "corona virus 2019" or "coronavirus 2019" or SARS-CoV-2 or "B.1.1.7" or "B.1.351" or "B.1.1.28" or "B.1.617" or "BA.1" or "BA.2" or "BA.3" or "BA.4" or "BA.5" or omicron or deltacron or "delta variant" or "delta subvariant" or "XBB.1.3") N2 (prolonged or "long haul*" or chronic or lingering or ongoing or persistent or "long term" or "more than 12 weeks" or "more than 24 weeks") ) OR AB ( (Covid or Covid19 or "corona virus 2019" or "coronavirus 2019" or SARS-CoV-2 or "B.1.1.7" or "B.1.351" or "B.1.1.28" or "B.1.617" or "BA.1" or "BA.2" or "BA.3" or "BA.4" or "BA.5" or omicron or deltacron or "delta variant" or "delta subvariant" or "XBB.1.3") N2 (prolonged or "long haul*" or chronic or lingering or ongoing or persistent or "long term" or "more than 12 weeks" or "more than 24 weeks") )
14. S11 OR S12 OR S13
15. S10 OR S14
16. (MH "Autoimmune Diseases")
17. TI ( (autoimmun* or auto immun*) and (disease* or disorder*) ) OR AB ( (autoimmun* or auto immun*) and (disease* or disorder*) )
18. (MH "Anemia, Hemolytic, Autoimmune")
19. TI ( Autoimmune hemolytic anemia* OR Autoimmune hemolytic anaemia* ) OR AB ( Autoimmune hemolytic anemia* OR Autoimmune hemolytic anaemia* )
20. (MH "Purpura, Thrombocytopenic")
21. TI Idiopathic thrombocytopenic purpura* OR AB Idiopathic thrombocytopenic purpura*
22. TI Cryoglobulinemia* OR AB Cryoglobulinemia*
23. (MH "Thyroiditis, Autoimmune") OR (MH "Graves' Disease")
24. TI ( Autoimmune Thyroiditi* or Autoimmune thyroid disease* or Grave* disease* or Hashimoto* Disease* or Hashimoto* thyroiditi* ) OR AB ( Autoimmune Thyroiditi* or Autoimmune thyroid disease* or Grave* disease* or Hashimoto* Disease* or Hashimoto* thyroiditi* )
25. (MH "Diabetes Mellitus, Type 1")
26. TI Type 1 N3 diabet* OR AB Type 1 N3 diabet*
27. (MH "Addison's Disease") OR (MH "Adrenal Insufficiency")
28. TI ( Addiso* disease* or Adrenal insufficienc* ) OR AB ( Addiso* disease* or Adrenal insufficienc* )
29. (MH "Multiple Sclerosis")
30. TI Multiple scleros* OR AB Multiple scleros*
31. (MH "Guillain-Barre Syndrome") OR (MH "Miller Fisher Syndrome")
32. TI Guillain-Barre-Syndrome* OR AB Guillain-Barre-Syndrome*
33. (MH "Myasthenia Gravis")
34. TI Myasthenia Gravis OR AB Myasthenia Gravis
35. (MH "Inflammatory Bowel Diseases") OR (MH "Crohn Disease") OR (MH "Colitis, Ulcerative")
36. TI ( Inflammatory Bowel Disease* or Ulcerative coliti* or Croh* Disease* or Morbus Crohn* ) OR AB ( Inflammatory Bowel Disease* or Ulcerative coliti* or Croh* Disease* or Morbus Crohn* )
37. (MH "Cholangitis")
38. TI Biliary cholangiti* OR AB Biliary cholangiti*
39. (MH "Hepatitis, Autoimmune")
40. TI Autoimmune hepatiti* OR AB Autoimmune hepatiti*
41. (MH "Celiac Disease")
42. TI ( Celiac disease* or coeliac disease* ) OR AB ( Celiac disease* or coeliac disease* )
43. (MH "Pemphigus")
44. TI Pemphigus vulgaris
45. (MH "Pemphigoid, Bullous")
46. TI Bullous pemphigoid
47. (MH "Dermatitis")
48. TI ( Dermatitis herpetiform* or Duhring* disease* ) OR AB ( Dermatitis herpetiform* or Duhring* disease* )
49. (MH "Psoriasis")
50. TI Psorias* OR AB Psorias*
51. (MH "Alopecia Areata") OR (MH "Alopecia")
52. TI Alopecia* OR AB Alopecia*
53. (MH "Vitiligo")
54. TI Vitiligo* OR AB Vitiligo*
55. (MH "Lupus Erythematosus, Cutaneous")
56. TI Cutaneous lupus erythemato* OR AB Cutaneous lupus erythemato*
57. (MH "Arthritis, Rheumatoid")
58. TI Rheumatoid arthriti* OR AB Rheumatoid arthriti*
59. (MH "Still's Disease, Adult-Onset")
60. TI Adult-onset Still* disease* OR AB Adult-onset Still* disease*
61. (MH "Vasculitis") OR (MH "Polyarteritis Nodosa") OR (MH "Takayasu Arteritis") OR (MH "Giant Cell Arteritis") OR (MH "Goodpasture's Syndrome")
62. TI ( Vasculiti* or Anti-Neutrophil Cytoplasmic Antibody-Associated Vasculiti* or ANCA-Associated Vasculiti* or Polyarteritis Nodos* or Anti-Glomerular Basement Membrane Disease* or Goodpasture* syndrome* or Takayasu arteriti* or Arteritis temporalis or Giant cell arteriti* or Temporal arteriti* or Cranial arteriti* ) OR AB ( Vasculiti* or Anti-Neutrophil Cytoplasmic Antibody-Associated Vasculiti* or ANCA-Associated Vasculiti* or Polyarteritis Nodos* or Anti-Glomerular Basement Membrane Disease* or Goodpasture* syndrome* or Takayasu arteriti* or Arteritis temporalis or Giant cell arteriti* or Temporal arteriti* or Cranial arteriti* )
63. (MH "Lupus Erythematosus, Systemic")
64. TI Systemic lupus erythemato* OR AB Systemic lupus erythemato*
65. (MH "Dermatomyositis") OR (MH "Polymyositis")
66. TI ( Dermatopolymyositi* or Dermatomyositi* or Polymyositi* ) OR AB ( Dermatopolymyositi* or Dermatomyositi* or Polymyositi* )
67. (MH "Scleroderma, Systemic")
68. TI Systemic sclero* OR AB Systemic sclero*
69. (MH "Sjogren's Syndrome")
70. TI Sjogren* syndrome* OR AB Sjogren* syndrome*
71. (MH "Connective Tissue Diseases")
72. TI Mixed connective tissue disease* OR AB Mixed connective tissue disease*
73. (MH "Polymyalgia Rheumatica")
74. TI Polymyalgia rheumatica OR AB Polymyalgia rheumatica
75. (MH "Spondylitis, Ankylosing")
76. TI Ankylosing spondylit* OR AB Ankylosing spondylit*
77. S16 OR S17 OR S18 OR S19 OR S20 OR S21 OR S22 OR S23 OR S24 OR S25 OR S26 OR S27 OR S28 OR S29 OR S30 OR S31 OR S32 OR S33 OR S34 OR S35 OR S36 OR S37 OR S38 OR S39 OR S40 OR S41 OR S42 OR S43 OR S44 OR S45 OR S46 OR S47 OR S48 OR S49 OR S50 OR S51 OR S52 OR S53 OR S54 OR S55 OR S56 OR S57 OR S58 OR S59 OR S60 OR S61 OR S62 OR S63 OR S64 OR S65 OR S66 OR S67 OR S68 OR S69 OR S70 OR S71 OR S72 OR S73 OR S74 OR S75 OR S76
78. S15 AND S77
79. S15 AND S77 **Limiters** - Publication Date: 20190101-20241231
80. TI ( (animal or animals or canine* or dog or dogs or feline or hamster* or lamb or lambs or mice or monkey or monkeys or mouse or murine or pig or pigs or piglet* or porcine or primate* or rabbit* or rats or rat or rodent* or sheep* ) NOT (human* or patient*)) **Limiters** - Publication Date: 20190101-20241231
81. S79 NOT S80 **Limiters** - Publication Date: 20190101-20241231
